# Long‐term outcomes of second‐line versus later‐line zanubrutinib treatment in patients with relapsed/refractory mantle cell lymphoma: An updated pooled analysis

**DOI:** 10.1002/cam4.6473

**Published:** 2023-09-14

**Authors:** Yuqin Song, Keshu Zhou, Dehui Zou, Dengju Li, Jianda Hu, Haiyan Yang, Huilai Zhang, Jie Ji, Wei Xu, Jie Jin, Fangfang Lv, Ru Feng, Sujun Gao, Daobin Zhou, Constantine S. Tam, David Simpson, Michael Wang, Tycel J. Phillips, Stephen Opat, Cheng Fang, Shaohui Sun, Jun Zhu

**Affiliations:** ^1^ Peking University Cancer Hospital and Institute Beijing China; ^2^ Affiliated Cancer Hospital of Zhengzhou University Henan Cancer Hospital Zhengzhou China; ^3^ Institute of Hematology & Blood Diseases Hospital, Chinese Academy of Medical Sciences and Peking Union Medical College Tianjin China; ^4^ Tongji Hospital, Tongji Medical College Huazhong University of Science and Technology Wuhan China; ^5^ Fujian Medical University Union Hospital Fuzhou China; ^6^ The Cancer Hospital of the University of Chinese Academy of Sciences (Zhejiang Cancer Hospital), Institute of Basic Medicine and Cancer (IBMC), Chinese Academy of Sciences Hangzhou China; ^7^ Tianjin Medical University Cancer Institute and Hospital Tianjin China; ^8^ West China Hospital of Sichuan University Chengdu China; ^9^ The First Affiliated Hospital of Nanjing Medical University, Jiangsu Province Hospital Nanjing China; ^10^ The First Affiliated Hospital Zhejiang University College of Medicine Hangzhou China; ^11^ Fudan University Shanghai Cancer Center Shanghai China; ^12^ Nanfang Hospital of Southern Medical University Guangzhou China; ^13^ The First Hospital of Jilin University Changchun China; ^14^ Peking Union Medical College Hospital Chinese Academy of Medical Sciences & Peking Union Medical College Beijing China; ^15^ Peter MacCallum Cancer Centre, St. Vincent's Hospital University of Melbourne Melbourne Victoria Australia; ^16^ North Shore Hospital Auckland New Zealand; ^17^ The University of Texas MD Anderson Cancer Center Houston Texas USA; ^18^ University of Michigan Ann Arbor Michigan USA; ^19^ Monash Health, Monash University Clayton Victoria Australia; ^20^ BeiGene (Beijing) Co., Ltd. Beijing China

**Keywords:** mantle cell lymphoma, overall survival, pooled analysis, second‐line therapy, zanubrutinib

## Abstract

**Background:**

We previously reported results of a pooled analysis of two zanubrutinib studies in relapsed or refractory (R/R) MCL showing better survival outcomes when zanubrutinib is used in second‐line versus later‐line. Here, we present an updated pooled analysis with a longer follow‐up of 35.2 months.

**Methods:**

Data were pooled from two studies—BGB‐3111‐AU‐003 (NCT02343120) and BGB‐3111‐206 (NCT03206970) of zanubrutinib in R/R MCL. The patients were divided into two groups based on the treatment line of zanubrutinib: the second‐line and the later‐line group. The inverse propensity score weighting method was used to balance the baseline covariates between the groups. The primary outcome was overall survival (OS). Secondary outcomes included progression‐free survival (PFS), PFS, and OS rates, objective response rate (ORR), duration of response (DOR), and safety.

**Results:**

Among 112 pooled patients, 41 (36.6%) patients received zanubrutinib as second‐line and 71 (63.4%) patients as later‐line therapy. After weighting, OS was significantly improved in the second‐line versus later‐line group (HR, 0.459 [95% CI: 0.215, 0.98]; *p* = 0.044) with median OS not estimable in both groups. The PFS was similar between the two groups (HR, 0.78 [95% CI: 0.443, 1.373]; *p* = 0.389) but with numerically longer median PFS in the second‐line versus later‐line group (27.8 vs. 22.1 months). ORR was numerically higher in the second‐line versus later‐line (88.6% vs. 85.7%), and DOR was similar between the two groups (25.2 vs. 25.1 months). Zanubrutinib showed a similar safety profile in both groups.

**Conclusion:**

Zanubrutinib in second‐line treatment was associated with significantly improved OS compared with later‐line treatment of R/R MCL.

## Abstract 2

- This pooled analysis of two studies of zanubrutinib examined its efficacy and safety in the treatment of patients with recurrent/relapsed mantle cell lymphoma (MCL) in two different settings—second‐line and later‐line with long‐term follow‐up.
- This analysis showed that patients receiving zanubrutinib in the second‐line had significantly better overall survival than those who received it in the later‐line.
- The finding suggests that earlier use of zanubrutinib in the treatment line may lead to better outcomes in patients with recurrent/relapsed MCL.

## 1 INTRODUCTION

Mantle cell lymphoma (MCL) is a rare, aggressive B‐cell non‐Hodgkin lymphoma with a poor prognosis and patients eventually relapse after frontline therapy.^1^, ^2^ Though relapsed or refractory (R/R) MCL is incurable, Bruton's tyrosine kinase (BTK) inhibitors have emerged as a new promising treatment strategy for these patients.^3^ Ibrutinib was the first BTK inhibitor approved in MCL and has shown convincing efficacy outcomes in these patients but is associated with off‐target side effects which could be treatment‐limiting.^4^ Zanubrutinib is a novel, second‐generation, irreversible BTK inhibitor with improved selectivity and less adverse events (AEs) than ibrutinib.^5^, ^6^ On the basis of a combined objective response rate (ORR) of 84% in 118 R/R MCL patients from two multicenter clinical trials, namely, BGB‐3111‐AU‐003 (NCT02343120) and BGB‐3111‐206 (NCT03206970), zanubrutinib, was approved by the US FDA in 2019 for treatment of R/R MCL.^7^, ^8^ A pooled analysis of these two studies also showed an ORR of 85% for zanubrutinib suggesting it to be a promising therapeutic option in R/R MCL.^9^

Though the long‐term outcome for patients with MCL has been improving, the published literature indicates that survival outcomes become worse with every successive line of chemoimmunotherapy.^10^ In general, median progression‐free survival (PFS) with chemoimmunotherapy has been reported to decline by 70% from first‐line (47.4 months) to second‐line (14.0 months) and by 86% after third‐line therapy (6.5 months). These facts along with new drugs, such as BTK inhibitors, becoming available for the treatment of R/R MCL, have led to a pressing need to prioritize and sequence these therapies in the treatment algorithm for attaining maximum and durable treatment responses. In this regard, there is a lack of adequate long‐term clinical data on the use of BTK inhibitors in R/R MCL for determining their appropriate place in treatment strategy. Hence, for BTK inhibitors in R/R MCL, studies are being conducted to gather more data to identify the right time and treatment line for initiating BTK inhibitor therapy in patients with R/R MCL for improved outcomes.

In a quest toward addressing these unmet needs, a pooled analysis of three ibrutinib studies involving R/R MCL patients had earlier reported better survival outcomes in patients receiving ibrutinib as second‐line therapy compared to those receiving it in late line setting (PFS: 25.4 months vs. 10.3 months; overall survival [OS]: 61.6 months vs. 22.5 months).^11^ Further, a pooled analysis of zanubrutinib pivotal studies BGB‐3111‐206 and BGB‐3111‐AU‐003 was done by segregating patients into those receiving zanubrutinib as second‐line therapy or later‐line therapy, and results showed zanubrutinib to be associated with numerically better PFS and OS when it was administered in the second‐line versus the later‐line in R/R MCL patients at a median follow‐up of 24.9 months, thereby suggesting the potential worthiness of identifying appropriate line of treatment with zanubrutinib.^9^ Here, we present a longer follow‐up (median 35.2 months) of the pooled datasets to compare long‐term outcomes of second‐line (with 1 prior line of therapy) versus later‐line (with >1 prior lines of therapy) zanubrutinib treatment in R/R MCL patients, which will also help to determine if the early start of BTK inhibitor monotherapy would benefit R/R MCL patients more than its use in later‐line.

## 2 METHODS

### 2.1 Study design and patients

This current analysis was an extension study with a longer follow‐up of a previously conducted pooled analysis involving R/R MCL patients treated with zanubrutinib in a phase I study (BGB‐3111‐AU‐003, NCT02343120) and a phase II study (BGB‐3111‐206, NCT03206970).^9^ Details of the two parent studies are given elsewhere.^7^, ^8^ The patients with no missing baseline covariates (age, sex, body mass index [BMI], Eastern Cooperative Oncology Group [ECOG] performance status, disease stage, blastoid variant, Mantle Cell Lymphoma International Prognostic Index [MIPI; defined as low (score <5.7), intermediate (score ≥5.7 and <6.5), and high (sore ≥6.5) risk], bulky disease [defined as the longest transverse diameter of a lesion >5 cm], extranodal and bone marrow involvement) were pooled. The patients were divided into two groups based on the line of treatment with zanubrutinib: the second‐line (with 1 prior line of therapy) and the later‐line group (with >1 prior lines of therapy).

Ethics committees or institutional review boards at the respective study sites approved the studies included in the analysis and which were conducted in compliance with the ethical principles of Good Clinical Practice, International Conference on Harmonization guidelines, the Declaration of Helsinki, and applicable local regulatory requirements.

### 2.2 Endpoints and assessments

The primary outcome was OS. Secondary outcomes included PFS, PFS rate, and OS rate at 12, 24, and 36 months, ORR, and duration of response (DOR). Response to treatment was assessed according to the Lugano classification.^12^ Positron emission tomography‐computed tomography (PET‐CT) scans were used to assess response at baseline and follow‐up in BGB‐3111‐206 study but were optional in BGB‐3111‐AU‐003 study where they were required only for confirmation of suspected CR. Therefore, due to the potential difference in the pooled CR rates that could be caused by the differential use of PET scan in two pooled studies, the CR rate was not compared between the second‐ and later‐line groups.

AEs were also assessed and coded using the Medical Dictionary for Regulatory Activities (MedDRA), version 20.0. The severity of AEs was graded according to the National Cancer Institute Common Toxicity Criteria (NCI CTCAE), version 4.03. AEs of special interest (AESI) were predefined as those known to be associated with BTK inhibitors and identified using predefined MedDRA version 20.0 search criteria. The selected AESI included hemorrhage (major hemorrhage defined as intracranial hemorrhage, severe hemorrhage, grade ≥3 bleeding from other tissues, or organs), atrial fibrillation and flutter, hypertension, and diarrhea.

### 2.3 Statistical analysis

The inverse propensity score weighting (IPSW) method was used to balance the baseline covariates (age, sex, BMI, ECOG, disease stage, blastoid variant, MIPI, bulky disease, extra‐nodal disease, and bone marrow involvement) between the second‐ and later‐line groups to mimic a randomized controlled trial.^13^, ^14^ For this pooled analysis, baseline covariates were used to create a propensity score model, along with the prior medication. The balance criteria for continuous variables were: (1) a standardized mean difference not exceeding 0.1; and (2) a ratio of variances between 0.67 and 1.5. The balance criterion for binary variables was an absolute mean difference not exceeding 0.1.^15^, ^16^ The IPSW process was designed to preserve the original prevalence of prior medication use after weighting.

Survival probability was estimated by the Kaplan–Meier method. The Cox proportional hazards model was used to evaluate the difference in PFS and OS between the groups and to compute the hazard ratio (HR) and 95% confidence interval (CI). All analyses were performed using R version 3.6.1 (WeightIt, survival and survey packages). This is a post hoc analysis and *p*‐values were reported for descriptive purposes.

## 3 RESULTS

### 3.1 Baseline characteristics

Overall, 112 patients with R/R MCL were included in this pooled analysis—33 from BGB‐3111‐AU‐003 study and 79 from BGB‐3111‐206 study; the median duration of follow‐up was 31.3 and 35.2 months, respectively. The median age (interquartile range) was 62 (54, 68) years with 41 (36.6%) patients aged ≥65 years. Most of the patients had Stage III or IV disease (*n* = 102; 91%) and 24 (21.4%) patients had high MIPI risk score. There were 67 (59.8%) patients with extranodal disease, 42 (37.5%) with bulky disease, and 14 (12.5%) with blastoid variant (Table S1).

Of 112 patients included in the study, 41 (36.6%) patients were in the second‐line group and 71 (63.4%) patients were in the later‐line group. Compared with the later‐line group, the second‐line group had a higher percentage of patients with age ≥ 65 years (46.3% vs. 31.0%) and MIPI high‐risk scores (26.8% vs. 18.3%) and lower percentages of patients with extranodal disease (51.2% vs. 64.8%), bulky disease (31.7% vs. 40.8%), and blastoid subtype (2.4% vs. 18.3%) (Table 1). After weighting, the baseline covariates were balanced between the second‐ and later‐line groups. The effective sample size was 91 patients of which 30 (33%) patients were in the second‐line group and 61 (67%) patients were in the later‐line group. In terms of prior treatment regimens, rituximab and RCHOP were used by 74% and 72% patients, respectively, in the second‐line group and by 79% and 89% patients, respectively, in the later‐line group. The percentage of patients who received prior bendamustine was low in both groups (4% in the second‐line and 5% in the later‐line) (Table 1).

**TABLE 1:** Baseline covariates in second‐line and later‐line groups before and after weighting.

Characteristic	Before weighting (*N* = 112)			After weighting (*N* = 91)		
	Second‐line group *N* = 41	Later‐line group *N* = 71	Mean difference (variance ratio)	Second‐line group *N* = 30	Later‐line group *N* = 61	Mean difference (variance ratio)
Age, median (IQR) years	64 (55, 71)	61 (54,66)	NA	61.5 (52.8, 67.2)	60.5 (53.2, 67.1)	NA
Mean (SD) years	63.2 (11.3)	60.6 (9.0)	0.26 (1.57)	61.0 (10.2)	61.2 (9.9)	−0.03 (1.06)
Age ≥ 65 years	19 (46.3%)	22 (31.0%)	NA	34%	35%	NA
Males	34 (82.9%)	52 (73.2%)	0.10	79%	77%	0.03
BMI, mean (SD)	25.4 (4.1)	24.7 (4.2)	0.17 (0.93)	24.6 (4.0)	24.8 (4.2)	−0.04 (0.88)
ECOG PS >1	2 (4.9%)	4 (5.6%)	−0.01	3%	4%	−0.01
Disease stage						
I	2 (4.9%)	1 (1.4%)	0.03	4%	4%	0.00
II	2 (4.9%)	5 (7.0%)	−0.02	5%	6%	−0.01
III	4 (9.8%)	10 (14.1%)	−0.04	18%	14%	0.05
IV	33 (80.5%)	55 (77.5%)	0.03	73%	76%	−0.03
Blastoid variant	1 (2.4%)	13 (18.3%)	−0.16	2%	12%	−0.10
Bulky disease^a^	13 (31.7%)	29 (40.8%)	−0.09	41%	39%	−0.02
Extra‐nodal disease	21 (51.2%)	46 (64.8%)	−0.14	63%	62%	0.01
MIPI						
Low risk	19 (46.3%)	36 (50.7%)	−0.04	53%	49%	0.04
Intermediate risk	11 (26.8%)	22 (31.0%)	−0.04	28%	30%	−0.02
High risk	11 (26.8%)	13 (18.3%)	0.09	19%	21%	−0.02
Refractory disease	26 (63.4%)	48 (67.6%)	−0.04	60%	71%	0.11
Prior medication use						
DHAP	22%	15%	NA	21%	19%	NA
Rituximab	80%	79%	NA	74%	79%	NA
RCHOP	73%	87%	NA	72%	89%	NA
Hyper CVAD	15%	20%	NA	12%	18%	NA
DICE	0	18%	NA	0	17%	NA
ASCT	5%	11%	NA	5%	11%	NA
Purine analogue	5%	17%	NA	11%	17%	NA
Lenalidomide	0	14%	NA	0	14%	NA
ESHAP	0	4%	NA	0	4%	NA
Bendamustine	5%	6%	NA	4%	5%	NA
Bortezomib	2%	10%	NA	1%	11%	NA

Abbreviations: ASCT, autologous stem cell transplantation; BMI, body mass index; DHAP, dexamethasone, high‐dose cytarabine and cisplatin; DICE, dexamethasone, isofosfamide, cisplatin, and etoposide; ECOG PS, Eastern Cooperative Oncology Group performance status; ESHAP, etoposide, methylprednisolone, high‐dose cytarabine and cisplatin; Hyper CVAD, cyclophosphamide, vincristine, doxorubicin and dexamethasone alternating with methotrexate and cytarabine; IQR, interquartile range; MIPI, Mantle Cell Lymphoma International Prognostic Index; RCHOP, rituximab, cyclophosphamide, doxorubicin, vincristine and prednisone; SD, standard deviation.

^a^ Defined as at least one lesion with longest diameter >5 cm.

### 3.2 Efficacy outcomes

#### 3.2.1 OS

**FIGURE 1:**
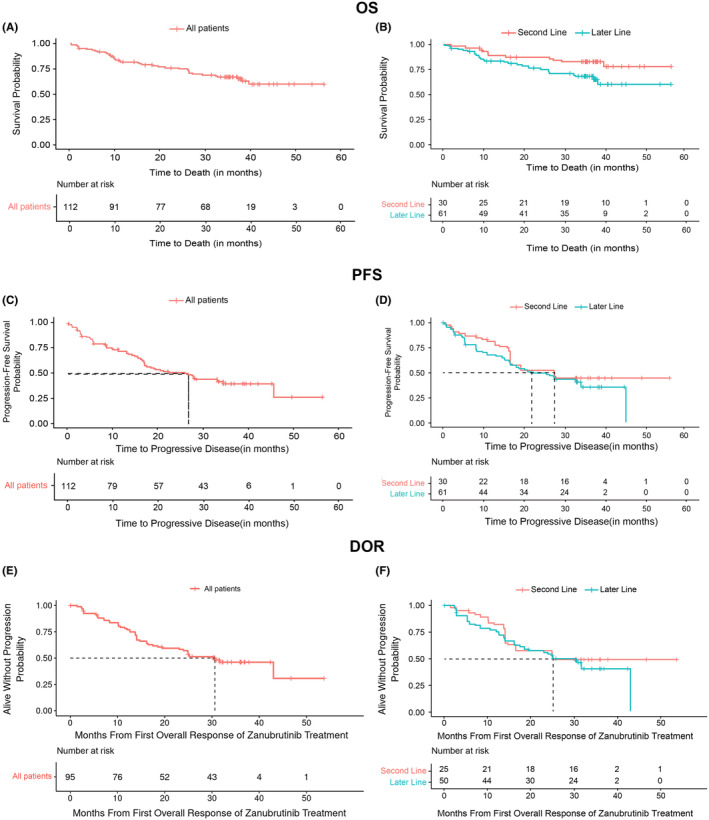
Overall survival (OS), progression‐free survival (PFS), and duration of response (DOR) in R/R MCL patients treated with zanubrutinib. OS: (A) overall pooled population, (B) by line of therapy after weighting; PFS: (C) overall pooled population, (D) by line of therapy after weighting; DOR: (E) overall pooled population, (F) by line of therapy after weighting.

In the overall pooled population, with a median follow‐up time of 35.2 months, the median OS was not estimable (NE). After weighting, OS was statistically significantly improved in the second‐line group versus the later‐line group (median OS: NE vs. NE; HR = 0.459; [95% CI: 0.215, 0.980]; *p* = 0.044) (Table 2 and Figure 1). In the original population (before weighting), OS was also numerically better in the second‐line group compared with the later‐line group (median OS: NE vs. NE; HR = 0.537 [95% CI: 0.276, 1.042]; *p* = 0.066) (Table 2 and Figure S1).

**TABLE 2:** Summary of efficacy outcomes before and after weighting.

	Before weighting (*N* = 112)			After weighting (*N* = 91)	
	Overall population (*N* = 112)	Second‐line group (*N* = 41)	Later‐line group (*N* = 71)	Second‐line group (ESS = 30)	Later‐line group (ESS = 61)
Median follow‐up, months	35.2	36.1	34.4	37.0	34.6
ORR, % (95% CI)	84.8 (76.8, 90.9)	87.8 (73.7, 95.9)	83.1 (72.3, 91.0)	88.6 (73.8, 96.6)	85.7 (75.6, 92.8)
Median DOR, months (95% CI)	30.6 (23.1, NE)	NE (14.7, NE)	30.6 (19.5, 43.1)	25.2 (14.1, NE)	25.1 (17.5, 43.0)
Median PFS, months (95% CI)	26.5 (16.8, NE)	27.8 (16.8, NE)	25.8 (16.7, 45.5)	27.8 (16.8, NE)	22.1 (16.6, 45.5)
Median OS, months (95% CI)	NE (39.6, NE)	NE (NE, NE)	NE (37.1, NE)	NE (NE, NE)	NE (38.2, NE)
PFS rate at, % (95% CI)					
12 months	72.4 (64.5, 81.3)	77.3 (65.8, 91.5)	69.6 (59.8, 81.4)	81.6 (70.7, 94.5)	68.0 (57.2, 81.7)
24 months	51.4 (42.7, 61.9)	53.3 (40.5, 71.9)	50.3 (40.1, 63.9)	52.4 (38.7, 74.3)	49.8 (39.1, 64.5)
36 months	40.3 (31.6, 51.4)	45.3 (32.9, 64.4)	36.8 (26.8, 52.0)	44.8 (31.8, 66.9)	35.4 (25.0, 52.3)
OS rate at, % (95% CI)					
12 months	81.8 (74.9, 89.3)	84.9 (74.8, 96.7)	79.9 (71.2, 89.9)	88.4 (79.8, 98.2)	82.6 (74.2, 92.1)
24 months	75.9 (68.2, 84.4)	82.3 (71.7, 95.1)	72.1 (62.5, 83.7)	86.4 (77.2, 97.0)	75.2 (65.7, 86.6)
36 months	66.8 (58.3, 76.5)	74.1 (62.0, 89.3)	62.4 (52.1, 75.5)	82.0 (71.7, 94.1)	66.5 (56.0, 79.5)

Abbreviations: CI, confidence interval; DOR, duration of response; ESS, effective sample sizes; NE, not estimable; ORR, objective response rate; OS, overall survival; PFS, progression‐free survival.

In the overall pooled population, the 36‐month OS rate was 66.8% (95% CI: 58.3%, 76.5%). After weighting, the 36‐month OS rate was numerically higher in the second‐line group (82.0% [95% CI: 71.7%, 94.1%]) than in the later‐line group (66.5% [95% CI: 56.0%, 79.5%]). A similar trend was also observed for second‐line versus later‐line group before weighting (74.1% [95% CI: 62.0%, 89.3%]) vs. 62.4% [95% CI: 52.1%, 75.5%]) (Table 2).

#### 3.2.2 PFS

The median PFS in the overall pooled population (before weighting) was 26.5 months (95% CI: 16.8, NE). After weighting, PFS was similar between the second‐line group and the later‐line group (HR = 0.78 [95% CI: 0.443, 1.373]; *p* = 0.389), with numerically longer median PFS in the second‐line group over the later‐line group (27.8 vs. 22.1 months) (Table 2 and Figure 1). Before weighting, PFS results were consistent with the post‐weighting results as the PFS was similar between the second‐line and later‐line groups (HR, 0.769 [95% CI: 0.459, 1.288]; *p* = 0.318) with numerically longer median PFS in the second‐line group than in the later‐line group (median PFS: 27.8 vs. 25.8 months) (Table 2 and Figure S1).

In the post‐weighting population, the 36‐month PFS rate was higher in the second‐line group (44.8% [95% CI: 31.8%, 66.9%)] than in the later‐line group (35.4% [95% CI: 25.0%; 52.3%]). A similar trend was also observed for two groups in before weighting population (Table 2).

#### 3.2.3 ORR

The ORR in the overall pooled population was 84.8% (95% CI: 76.8%, 90.9%) (Table 2). After weighting, the ORR was numerically higher in the second‐line group than in the later‐line group (88.6% vs. 85.7%). In before weighting population also, the ORR was higher in the second‐line group than in the later‐line group (87.8% vs. 83.1%) (Table 2).

#### 3.2.4 DOR

The median DOR in the overall pooled population was 30.6 months (95% CI: 23.1, NE) (Table 2). After weighting, the second‐line and later‐line groups showed a similar DOR (25.2 vs. 25.1 months) (Table 2 and Figure 1). The DOR was not yet estimable in the second‐line group versus 30.6 months in the later‐line group before weighting (Table 2 and Figure S1).

#### 3.2.5 Safety

The overall exposure duration of treatment in the pooled population was 22.8 months, with the second‐line group having a longer duration versus the later‐line group (before weighting: 24.3 vs. 21.9 months and after weighting: 25.1 vs. 21.6 months) (Table 3). Overall, around 96.4% of patients experienced at least one AE and 53.6% had at least one grade ≥3 AE (Table 3). In the post‐weighting population, 94.6% and 98.2% of patients in second‐line and later‐line, respectively, experienced at least one AE, while 56.9% and 47.8% of patients, respectively had a grade ≥3 AE (Table 3).

**TABLE 3:** Extent of zanubrutinib exposure, adverse events, and treatment status before and after weighting.

	Before weighting			After weighting	
	Second‐line group (*N* = 41)	Later‐line group (*N* = 71)	Total (*N* = 112)	Second‐line group (ESS = 30)	Later‐line group (ESS = 61)
Duration of treatment (months)	24.3	21.9	22.8	25.1	21.6
Adverse events					
At least one AE, %	95.1	97.2	96.4	94.6	98.2
At least one grade ≥3 AE, %	58.5	50.7	53.6	56.9	47.8
Grade 5 AE, %	4.9	9.9	8.0	3.0	8.3
Dose reduction due to AE, %	4.9	4.2	4.5	3.6	3.6
Dose interruption due to AE, %	7.3	14.1	11.6	7.6	11.6
Treatment discontinuation, %					
Due to AE, %	17.1	9.9	12.5	10.6	10.0
Due to PD, %	39.0	52.1	47.3	44.5	50.9
Due to withdrawal, %	0	1.4	0.9	0	3.4
Due to investigators, %	0	1.4	0.9	0	1.0

Abbreviations: AE, adverse events, ESS, effective sample size; PD, progressive disease.

In before weighting population, grade ≥3 AESI occurred in 39% of patients. After weighting, the percentages of patients with grade ≥3 AESI were 44% in the second‐line group and 33% in the later‐line group (Table 3). The grade ≥3 atrial fibrillation/flutter was low in both groups (1% in second‐line and 2% in later‐line). Further, grade ≥3 hypertension was observed in 4% and 2% of patients in second‐line and later‐line, respectively, and grade ≥3 hemorrhage in 1% and 3% of patients, respectively (Table 4).

**TABLE 4:** Adverse events of special interest in before and after weighting.

	Overall population (*N* = 112)		Before weighting (*N* = 112)				After weighting (*N* = 91)			
	Any grade	Grade ≥3	Second‐line any grade (*N* = 41)	Second‐line grade ≥3 (*N* = 41)	Later‐line any grade (*N* = 71)	Later‐line grade ≥3 (*N* = 71)	Second‐line any grade (*N* = 30)	Second‐line grade ≥3 (*N* = 30)	Later‐line any grade (*N* = 61)	Later‐line grade ≥3 (*N* = 61)
AESI										
At least one AESI, %	88	39	80	44	92	37	82	44	92	33
At least one atrial fibrillation/flutter, %	3	2	5	2	1	1	2	1	2	2
At least one diarrhea, %	25	1	27	0	24	1	21	0	26	1
At least one hemorrhage, %	5	4	2	2	7	4	1	1	6	3
At least one hypertension, %	12	3	15	2	11	3	16	4	12	2

Abbreviation: AESI, adverse events of special interest.

Overall, the safety profile in second‐line and later‐line groups were similar in both before‐weighting and post‐weighting populations (Tables 3 and 4).

## 4 DISCUSSION

The available therapies for R/R MCL are not curative with conventional therapy, their goal being to palliate and prolong survival. BTK inhibitors changed the treatment landscape of R/R MCL. Despite the impressive results of deep remission in long‐term treatment with BTK inhibitors in R/R MCL patients,^17^ the data on long‐term survival outcomes with these inhibitors in the appropriate line of treatment are limited.

We have previously shown convincing efficacy and safety data of zanubrutinib, a novel second‐generation BTK inhibitor in BGB‐3111‐AU‐003 and BGB‐3111‐206 studies in R/R MCL patients. An ORR of 84.8% was obtained after a pooled analysis of these two studies at a median follow‐up of 24.9 months and treatment duration of 20.4 months.^9^ The analysis also revealed a tendency of improved survival outcomes in R/R MCL treated with zanubrutinib in the second‐line as compared with zanubrutinib in the later‐line setting. In this current extended follow‐up analysis at 35.2 months, we demonstrated the continued efficacy of single‐agent zanubrutinib in patients with R/R MCL in the overall pooled population along with further evidence of better clinical performance when the drug is used after the first relapse rather than later relapses.

PFS is used as the surrogate endpoint of OS in some studies with a shorter duration of follow‐up.^18^ However, OS remains the gold standard as the most relevant endpoint for the measurement of survival.^19^ As this pooled analysis had a relatively long follow‐up period of 35.2 months, OS was used as the primary endpoint as it holds more clinical relevance here and could help in establishing the clinical benefit of earlier use of zanubrutinib in patients with R/R MCL. After balancing, a significantly longer OS was achieved in the second‐line group versus the later‐line group (HR = 0.459; *p* = 0.044), with the median OS of NE in both groups. The 36‐month OS rate was also higher in the second‐line group (82.0% vs. 66.5%). Though median OS was NE for both the groups before weighting also, the difference was not statistically significant; nevertheless, there was still a tendency for better OS in the second‐line than in the later‐line with a higher number of patients achieving OS at 36‐month in second‐line group (74.1% vs. 62.4%). These results indicate that if the covariates/characteristics are balanced between the two groups, OS is better in the second‐line versus later‐line treatment with zanubrutinib. Further, the median PFS shortened as the treatment lines increased. The median PFS in the second‐line group was 27.8 months, which was numerically longer than 22.1 months observed in the later‐line group (36 months rate: 44.8% vs. 35.4%). Supporting these findings, an exploratory analysis of a phase 3 R/R MCL study of ibrutinib (RAY) also depicted similar trends of better survival outcomes in patients given ibrutinib earlier in treatment.^20^, ^21^ The results from our study were also in line with a previously published pooled analysis of three studies of ibrutinib monotherapy in R/R MCL (PCYC‐1104, RAY, and SPARK) that showed a prolonged OS and PFS in patients with second‐line therapy compared to patients with later‐line of therapy at 9.7 years follow‐up.^11^

All these results showed more sustained and durable survival outcomes over long‐term follow‐ups in R/R MCL patients treated with BTK inhibitors in the early line than in the later lines. These findings hold considerable importance as it is known that the survival outcomes in MCL become worse with every relapse and increasing line of treatment.^22^ The previous studies have indicated that the earlier initiated treatment of MCL patients could lead to a survival benefit. Therefore, the optimal place for the initiation of BTK inhibitor therapy in the treatment of patients with R/R MCL to maximize the survival benefits remains a matter of interest.

Besides convincing survival outcomes, no new safety signals were recorded in this study, and the AEs observed in both second‐line and later‐line zanubrutinib treatment groups were consistent with the known safety profile of zanubrutinib in clinical setting. Notably, the frequency of potential grade ≥3 cardiovascular AESI of atrial fibrillation/flutter (second‐line: 1%; later‐line: 2%) and hypertension (second‐line: 4%; later‐line: 2%) reported were low and comparable to previously published reports indicating zanubrutinib to be a safe treatment option in the treatment of R/R MCL.^7^, ^9^

A notable aspect of this pooled analysis was the use of IPSW to balance the baseline characteristics between the two groups. As IPSW can help summarize all patient characteristics to a single covariate, it is a preferred method used in studies with a large number of confounders or a small number of events. Further, IPSW is also a better method in terms of retaining the patients in an analysis thereby increasing the effective sample size compared to other methods.^23^

This study may be limited by the nature of the design as a post hoc analysis, its small sample size, and the single‐arm design of the parent studies used for pooling the patient population. Nevertheless, it adds important data to the growing body of evidence supporting the use of BTK inhibitors in the early line of treatment after relapse rather than later lines and holds promise to help clinicians make more informed decisions in the management of R/R MCL.

## 5 CONCLUSION

Overall, the results from this 35.2‐month follow‐up analysis confirm the long‐term efficacy and safety of zanubrutinib monotherapy in R/R MCL. The study also confirmed that second‐line zanubrutinib treatment was associated with significantly improved OS compared with later‐line treatment in patients with R/R MCL along with a similar well‐tolerated safety profile, thereby supporting the earlier use of zanubrutinib in the treatment setting. Subsequent prospective studies with larger sample size will further help define the optimal position of zanubrutinib in the treatment sequence of R/R MCL. Concluding, zanubrutinib is an effective and well‐tolerated therapeutic option for R/R MCL and an early treatment with zanubrutinib tends to have better survival outcomes.

## AUTHOR CONTRIBUTIONS

**Yuqin Song:** Conceptualization (equal); data curation (equal); investigation (equal); writing – review and editing (equal). **Keshu Zhou:** Data curation (equal); investigation (equal); writing – review and editing (equal). **Dehui Zou:** Data curation (equal); investigation (equal); writing – review and editing (equal). **Dengju Li:** Data curation (equal); investigation (equal); writing – review and editing (equal). **Jianda Hu:** Data curation (equal); investigation (equal); writing – review and editing (equal). **Haiyan Yang:** Data curation (equal); investigation (equal); writing – review and editing (equal). **Huilai Zhang:** Data curation (equal); investigation (equal); writing – review and editing (equal). **Jie Ji:** Data curation (equal); investigation (equal); writing – review and editing (equal). **Wei Xu:** Data curation (equal); investigation (equal); writing – review and editing (equal). **Jie Jin:** Data curation (equal); investigation (equal); writing – review and editing (equal). **Fangfang Lv:** Data curation (equal); investigation (equal); writing – review and editing (equal). **Ru Feng:** Data curation (equal); investigation (equal); writing – review and editing (equal). **Sujun Gao:** Data curation (equal); investigation (equal); writing – review and editing (equal). **Daobin Zhou:** Data curation (equal); investigation (equal); writing – review and editing (equal). **Constantine S Tam:** Data curation (equal); investigation (equal); writing – review and editing (equal). **David Simpson:** Data curation (equal); investigation (equal); writing – review and editing (equal). **Michael Wang:** Data curation (equal); investigation (equal); writing – review and editing (equal). **Tycel J Phillips:** Data curation (equal); investigation (equal); writing – review and editing (equal). **Stephen Opat:** Data curation (equal); investigation (equal); writing – review and editing (equal). **Cheng Fang:** Project administration (equal); writing – original draft (equal); writing – review and editing (equal). **Shaohui Sun:** Formal analysis (equal); methodology (equal); software (equal); validation (equal); writing – review and editing (equal). **Jun Zhu:** Conceptualization (equal); data curation (equal); investigation (equal); supervision (equal); writing – review and editing (equal).

## CONFLICT OF INTEREST STATEMENT

Cheng Fang and Shaohui Sun are employees of BeiGene Co., Ltd., Beijing, China. All other authors declare no competing interests.

## ETHICS STATEMENT

Ethics committees or institutional review boards at the respective study sites approved the studies included in the analysis and which were conducted in compliance with the ethical principles of Good Clinical Practice, International Conference on Harmonization guidelines, the Declaration of Helsinki, and applicable local regulatory requirements.

## PATIENT CONSENT STATEMENT

Not applicable.

## Supporting information


Figure S1.

Table S1.

## Data Availability

The datasets used and/or analyzed during the current study are available from the corresponding author upon reasonable request.
