# Supplementary figures and images for: Long‐term outcomes of second‐line versus later‐line zanubrutinib treatment in patients with relapsed/refractory mantle cell lymphoma: An updated pooled analysis
Source: Cancer Med. 2023 Sep 14;12(18):18643–53. doi: 10.1002/cam4.6473 (PMC10557885; doi:10.1002/cam4.6473)

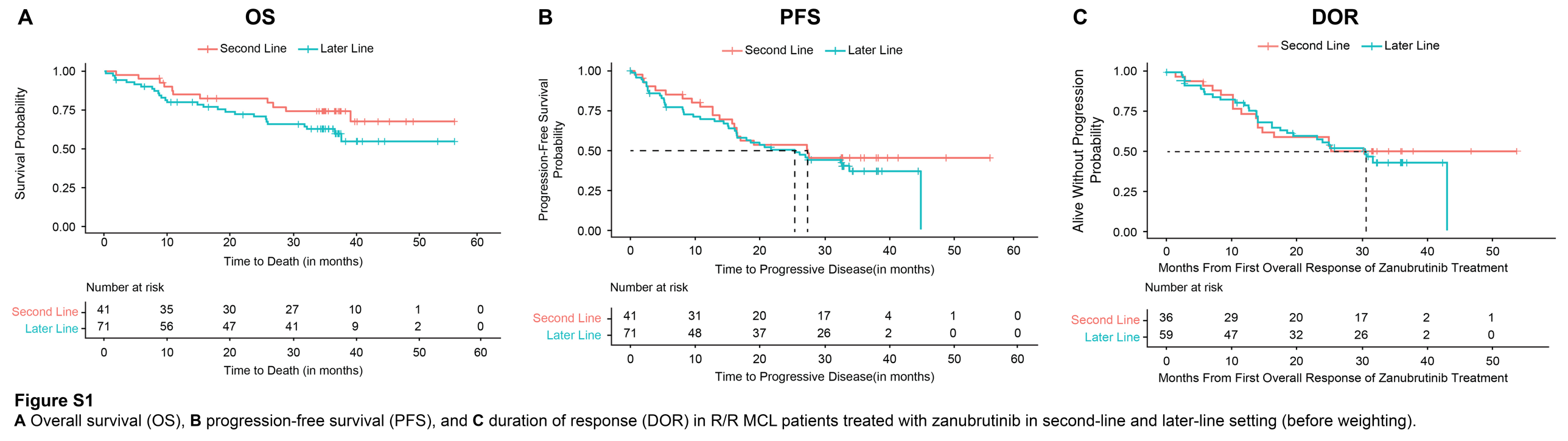

Supplement: Supplementary file 1 — Figure S1. [file CAM4-12-18643-s002.jpg]
