# Supplementary material for: Long‐term outcomes of second‐line versus later‐line zanubrutinib treatment in patients with relapsed/refractory mantle cell lymphoma: An updated pooled analysis
Source: Cancer Med. 2023 Sep 14;12(18):18643–53. doi: 10.1002/cam4.6473 (PMC10557885; doi:10.1002/cam4.6473)
Supplement: Supplementary file 2 — Table S1. [file CAM4-12-18643-s001.docx]

**Supplementary data**

**Table S1. Baseline covariates in two parent trials**

| Characteristic | Overall population | BGB-3111-AU-003 (N=33) | BGB-3111-206 (N=79) |
| --- | --- | --- | --- |
| Age, median (IQR) | 62.0 (54.0, 68.0) | 70.0 (64.0, 77.0) | 60.0 (53.5, 64.0) |
| Age ≥65 years, n (%) | 41 (36.6) | 24 (72.7) | 17 (21.5) |
| Males, n (%) | 86 (76.8) | 25 (75.8) | 61 (77.2) |
| BMI, mean (SD) | 24.9 (4.2) | 27.9 (4.8) | 23.7 (3.2) |
| ECOG PS >1 n (%) | 6 (5.4) | 3 (9.1) | 3 (3.8) |
| Disease stage, n (%) |  |  |  |
| I | 3 (2.7) | 2 (6.1) | 1 (1.3) |
| II | 7 (6.2) | 0 (0.0) | 7 (8.9) |
| III | 14 (12.5) | 1 (3.0) | 13 (16.5) |
| IV | 88 (78.6) | 30 (90.9) | 58 (73.4) |
| Blastoid variant, n (%) | 14 (12.5) | 2 (6.1) | 12 (15.2) |
| Bulky disease^*^, n (%) | 42 (37.5) | 7 (21.2) | 35 (44.3) |
| Extra-nodal disease, n (%) | 67 (59.8) | 9 (27.3) | 58 (73.4) |
| MIPI, n (%) |  |  |  |
| Low risk | 55 (49.1) | 8 (24.2) | 47 (59.5) |
| Intermediate risk | 33 (29.5) | 10 (30.3) | 23 (29.1) |
| High risk | 24 (21.4) | 15 (45.5) | 9 (11.4) |
| Refractory disease, n (%) | 74 (66.1) | 33 (100.0) | 41 (51.9) |

^*^Defined as at least one lesion with longest diameter >5 cm.

Abbreviations: BMI, body mass index; ECOG PS, Eastern Cooperative Oncology Group performance status; IQR, interquartile range; MIPI, Mantle Cell Lymphoma International prognostic index; SD, standard deviation
